# Pandemic influenza dynamics and the breakdown of herd immunity

**DOI:** 10.1371/currents.RRN1046

**Published:** 2009-10-01

**Authors:** Guy Katriel, Lewi Stone

**Affiliations:** ^*^Biomathematics Unit, Faculty of Life Sciences, Tel Aviv University and ^†^Tel Aviv University

## Abstract

Few if any attempts have been made to derive forecasts for the ongoing H1N1 pandemic as extrapolated from knowledge of seasonal influenza. Even simple back-of-the-envelope calculations are lacking. In this note we use first-approximation parameter estimates for the SIR model to compare seasonal and pandemic influenza, and then explore the implications of the existing classical epidemiological theory. In particular, we note the dramatic nonlinear increase in attack rate as a function of the percentage of susceptibles initially present in the population. This has severe consequences for the pandemic, given the general lack of immunity in the global population.

While the current understanding of the dynamics of seasonal influenza is far from complete, it is certainly at a more advanced level than that for the new influenza A H1N1 pandemic now sweeping across the globe [1]. Despite this, few if any attempts have been made to derive forecasts for the pandemic as extrapolated from knowledge of seasonal influenza. Even basic back-of-the-envelope calculations are lacking. In this note we explore the implications of the existing classical epidemiological theory.

                              Three characteristic properties specific to seasonal influenza are drawn upon (Appendix): a) The attack rate, or percentage of a region's population infected over the entire influenza season, lies somewhere between 5-15%.  For convenience, the attack rate is approximated as 10%.  b)  The duration of seasonal influenza is approximately three months.  c) The average infectious period of a sick individual is 3 days. By virtue of these three pieces of information, elementary calculations based on the classical SIR epidemic model show that the initial susceptibility of the population must lie in the vicinity of \begin{equation*}S_0=0.32\end{equation*} (see Appendix). That is, at the beginning of the season some one third of the population is susceptible and has the potential to be infected. This level of susceptibility seems reasonable given that a large component of the population has most likely gained immunity from previous exposure to related strains of the current influenza virus. 

                           The same calculations show that the value of the basic reproductive number \begin{equation*}R_0\end{equation*} [2], which describes the number of infections generated by a typically infected individual in a wholly susceptible population, is approximately \begin{equation*}R_0=3.75\end{equation*} for seasonal flu. The effective reproductive number \begin{equation*}R_e\end{equation*}, which describes the number of infections generated by an infected individual in the partially susceptible population (see eg., [3] [4]) at the beginning of the epidemic, is thus estimated as. \begin{equation*}R_e(seasonal)=0.32*3.75=1.2\end{equation*}. 

Consider now the arrival of pandemic influenza into a region. In the absence of previous exposure to the pandemic, it is reasonable to assume that a much larger proportion of the population is susceptible than is the case for the seasonal flu. Our working hypothesis is that there are no other relevant epidemiological differences between the two types of influenza. In particular, we assume that \begin{equation*}R_0(pandemic)=R_0(seasonal)=3.75\end{equation*}.

When modeling pandemic influenza and estimating \begin{equation*}R_0\end{equation*}, it is often taken for granted that the entire population is susceptible. This assumption, however, has been shown to be questionable [3] [4]. Indeed Mathews et al. (personal communication), based on model-fitting, have estimated that in the 1918 pandemic in the UK only 52% (95% confidence interval 41-66%) of the population were susceptible. Moreover, we argue that by considering the expected duration of an epidemic, calculations based on the SIR model indicate that 100%  population susceptibility is unlikely (Appendix). 

In the following we begin by assuming there is 64% susceptibility for pandemic influenza, which is twice that we use for seasonal influenza. How then does this simple difference in population susceptibility change the influenza attack rate? A naïve approach might suggest that if there are twice as many more susceptibles in the population, the attack rate for the pandemic might be expected to be 20% of the population instead of 10% for seasonal influenza. 

Although well established in epidemiological theory, it is often unappreciated that the attack rate of an epidemic is not determined by \begin{equation*}R_0\end{equation*} alone. The calculation (as based on the SIR model) involves two stages: i) Determining the fraction \begin{equation*}Z\end{equation*} of susceptibles who become infected during an epidemic as the solution of the “final size equation" [5] \begin{equation*}1-Z=e^{-R_e Z}\end{equation*}, where \begin{equation*}R_e\end{equation*}  is the effective reproduction number. ii) Calculating the attack rate as given by \begin{equation*}A=S_0\cdot Z\end{equation*}.

 Since we assume that \begin{equation*}S_0\end{equation*}    is twice as high for the pandemic influenza as for the seasonal flu, we obtain that \begin{equation*}R_e(pandemic)=2\cdot R_e(seasonal)=2\cdot 1.2=2.4\end{equation*}. From (i&ii) above, a larger value of \begin{equation*}R_e\end{equation*}  increases the size of the epidemic in two ways.  Firstly, the quantity ***Z***, the fraction of susceptibles who become infected, is much larger. Solving for \begin{equation*}R_e=2.4\end{equation*} , we obtain \begin{equation*}Z=0.88\end{equation*} (that is - %88 of susceptibles will be infected during the pandemic), in contrast to  \begin{equation*}Z=0.31\end{equation*} for seasonal flu. Secondly, there are more susceptibles so that the attack rate \begin{equation*}A=S_0\cdot Z=0.88*0.64=0.58\end{equation*}. Thus although the estimated number of susceptibles for the pandemic is twice that for the seasonal flu, the resulting attack rate is 5.8 times higher (and 2.9 times higher than the “naïve” prediction).

It is interesting to note that the naïve prediction is based on the supposition that doubling the number of susceptibles should double the number of people infected. However the flaw in this logic derives from a collective phenomenon whereby for low levels of susceptibles the population inherits a protection akin to herd immunity [2]. That is, large numbers of immune individuals tend to block infection routes and thereby reduce the risks of infection for the entire population. Thus increasing the number of susceptibles leads to a breakdown in herd immunity and effectively amplifies the risks of the epidemic to levels well beyond the naïve prediction. This is demonstrated in Figure 1 which displays a graph of the true attack rate as a function of \begin{equation*}S_0\end{equation*} and provides a comparison with the naïve prediction.

It should be stressed that the above estimates are subject to the uncertainty in the estimate of the attack rate of seasonal influenza (assumption (a)) as well as to uncertainty  in the actual fraction of the population susceptible to the pandemic influenza (which we took to be  \begin{equation*}S_0=0.64\end{equation*}). Nevertheless, the goal here is not to give exact predictions but to convey the conceptual mechanisms involved, and sometimes the nonintuitive outcomes when a pandemic triggers. Most significantly, the epidemic attack rate can reach unexpectedly high levels. While the above SIR modeling analysis provides an outline of the processes at work, it obviously does not take into account a number of subtleties and complexities characteristic to influenza dynamics in heterogeneous populations. However, given that the simple model is known for its unusual robustness, the conclusions reported here regarding the influenza A H1N1 pandemic demand serious attention and at the very minimum serve as a base-line for stimulating  future debate around these important issues.

**Figure:**
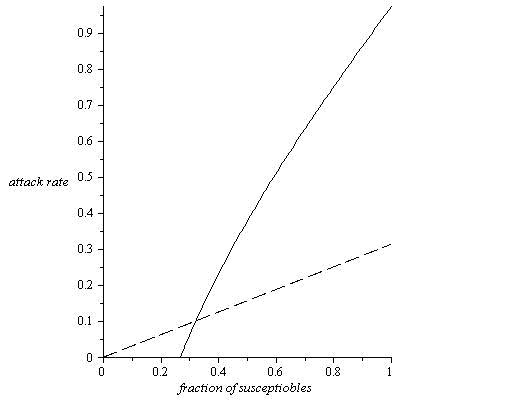
Figure 1.   Assuming \begin{equation*}R_0=3.75\end{equation*}, the attack rate (continuous line) is plotted as a function of the initial fraction \begin{equation*}S_0\end{equation*} of susceptibles in the population. An epidemic will not trigger unless the initial susceptibles are greater than****** \begin{equation*}S_0=0.27\end{equation*}, due to herd immunity.    The dashed line shows the naïve prediction for the attack rate,  obtained by extrapolating linearly from the %10 attack rate for \begin{equation*}S_0=0.32\end{equation*}, which can be well below the theoretical estimate.

## Appendix

## Funding information

 We acknowledge the support of EU-FP7 grant Epiwork, the Israel Science Foundation and the Israel Ministry of Health.

## Competing interests

 The authors have declared that no competing interests exist. 
